# A Stellar Case of Splenic Hydatid Cyst

**DOI:** 10.7759/cureus.63372

**Published:** 2024-06-28

**Authors:** Rishika Bhatnagar, Snehlata Hingway, Priya B Chatterjee

**Affiliations:** 1 Pathology, Jawaharlal Nehru Medical College, Datta Meghe Institute of Higher Education and Research, Wardha, IND

**Keywords:** echinococcus granulosus larvae, hydatid cyst of spleen, splenectomy, zoonotic disease, echinococcus granulosus, hydatid cyst, spleen

## Abstract

Hydatid disease, also known as hydatidosis or echinococcosis, is a zoonotic infection caused by cestode, namely *Echinococcus granulosus* (tapeworm). Humans are the incidental hosts that acquire the infection by being in contact with infected animals or through the fecal-oral route via contaminated feces. Hydatid disease of the spleen is a zoonotic disease of rare occurrence. Most often, the patients do not have any specific symptoms except dull dragging pain in the abdomen. In some unfortunate cases, the patient may present with an acute abdomen or anaphylactic shock state due to rupture of the cyst, which is a medical and surgical emergency. The mainstay of treatment remains albendazole and praziquantel medically, along with surgery, i.e., splenectomy. A 30-year-old female presented in the OPD with complaints of pain in the abdomen for the last two years with no other complaints. The pain did not respond to regular analgesics and antacids. The patient was admitted for further evaluation. A contrast-enhanced computed tomography (CECT) abdomen was done for the patient, which showed splenomegaly along with features suggestive of a splenic hydatid cyst. The lady was taken for a planned splenectomy. The histopathological features were suggestive of a hydatid cyst of the spleen. The mainstay of treatment is medically anthelmintic medications and surgical splenectomy along with the puncture aspiration injection re-aspiration (PAIR) technique.

## Introduction

Hydatid disease, also known as echinococcosis, is a parasite infestation caused by a cestode, namely *Echinococcus granulosus*, also known as tapeworm. Organs typically involved by Echinococcus are most commonly the liver (75%), followed by lungs (15%) and spleen (5%) [1]. Though the occurrence of hydatid disease is common in endemic areas, the incidence of infestations that involve the spleen, which is the primary organ, is infrequent. 

Pathophysiologically, humans are the incidental hosts of *E. granulosus* (hosts that have no role in the transmission cycle), with dogs being the definitive hosts (hosts that are part of the reproductive cycle of the parasite).

In myriad cases, patients do not have any suspicious symptoms. However, some symptoms may be present, such as dull aching pain, not exaggerated or alleviated by food or analgesics. Due to an increase in the size of the spleen, pressure symptoms may be present like dyspepsia, dyspnea, i.e., difficulty in breathing due to pressure exerted by the spleen on the right side of the diaphragm, constipation due to the pressure on the colon. In some gruesome cases, the patient may be directly present in the state of anaphylaxis [2]. Due to rupture of the cyst, which is a medical as well as surgical emergency.

With such an extremely low rate of incidence, hydatid cysts in the spleen are a sporadic pathology. To have come across this case of a 30-year-old female with no symptoms except for dull aching pain in the abdomen (more evident in the left hypochondrium) and to have reached the diagnosis of a hydatid cyst of the spleen was a sheer good chance. Hence, we report this case for an easier further evaluation of hydatidosis of the spleen by fellow clinicians and pathologists.

## Case presentation

**Figure 1:**
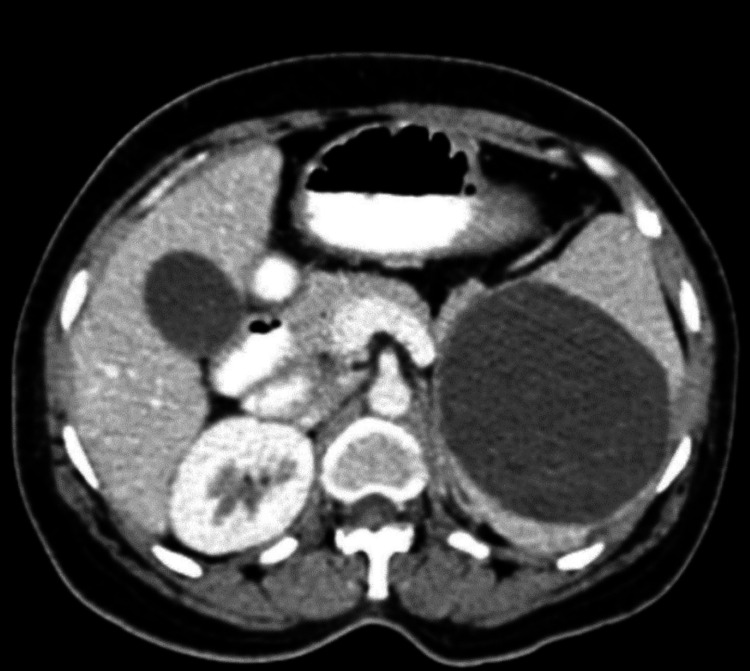
CECT image showing splenomegaly along with features of splenic hydatid cyst. CECT: contrast-enhanced computed tomography.

A 30-year-old female presented in the OPD with complaints of pain in the abdomen for the last two years with no other complaints. Pain was continuous in nature, non-radiating, and did not have any aggravating or relieving factors. It was mild and constant. There was no associated fever, history of weight loss, change in bowel patterns, change in eating patterns, or dyspepsia. The pain did not respond to regular analgesics and antacids. On examination, she had tenderness in the left upper quadrant, raising suspicion for splenic etiology. The patient was admitted for further evaluation. On admission, the vitals were normal. Hematological investigations were sent, which were within normal limits. A contrast-enhanced computed tomography (CECT) abdomen was done for the patient, which showed splenomegaly along with features suggestive of a splenic hydatid cyst (Figure 1). The lady was taken for a planned splenectomy after receiving clearance in the pre-anesthetic check-up. The surgery was uneventful. Specimen of the spleen was sent for histopathology for confirmation of the diagnosis. Postoperatively, there was thrombocytosis ranging from 6,21,000 to 7,76,000 cells/cumm. Medical opinion for the same was taken, and the patient was out on Ecosprin 75 mg in view of thrombocytosis status post-splenectomy with monitoring of platelet count every alternate day.

**Figure 2:**
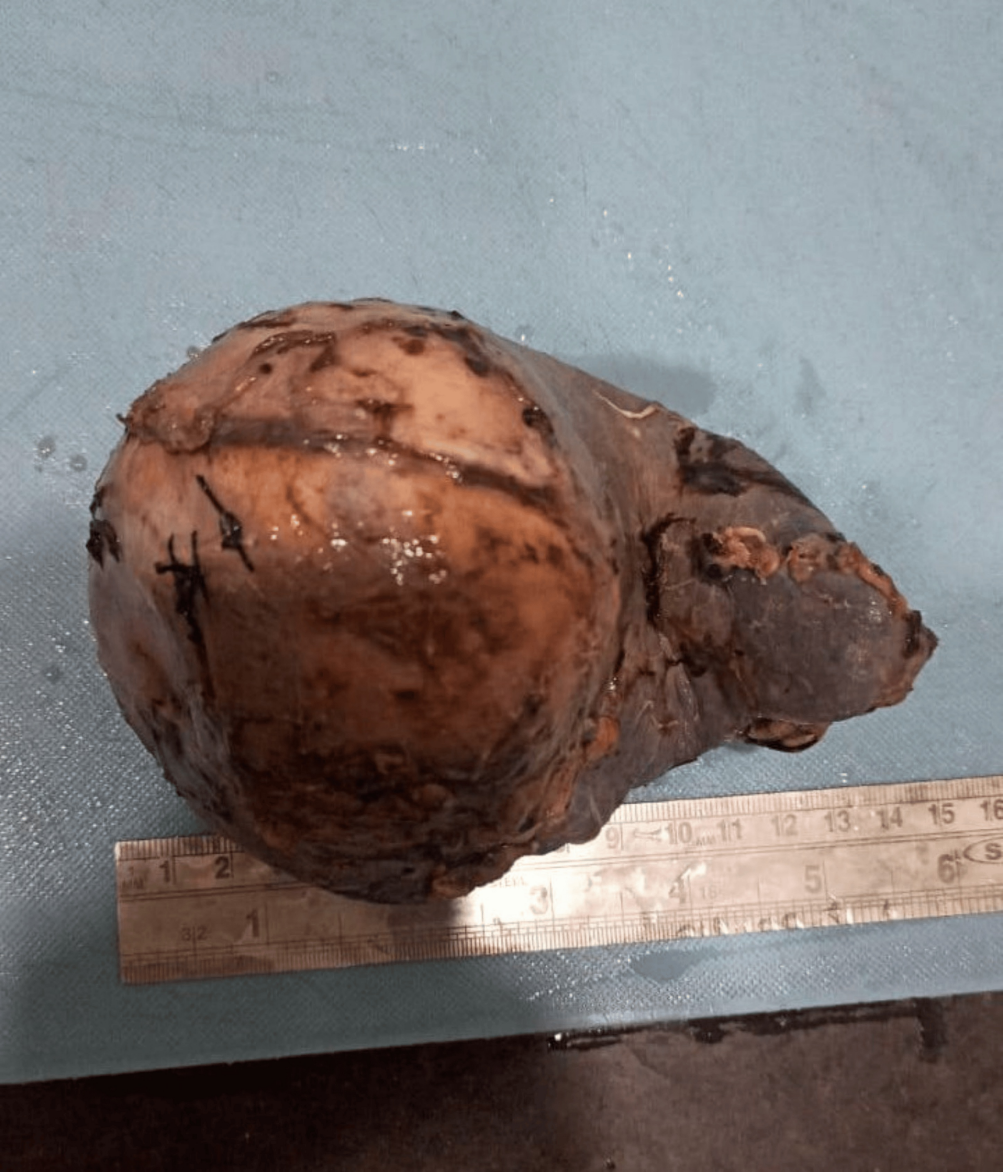
Spleen measuring 15 x 10 x 5.5 cm covered by capsule.

In histopathology, post-splenectomy, the specimen of the spleen was received. On gross examination, the spleen was measuring 15 x 10 x 5.5 cm and was covered by a capsule (Figure 2).

**Figure 3:**
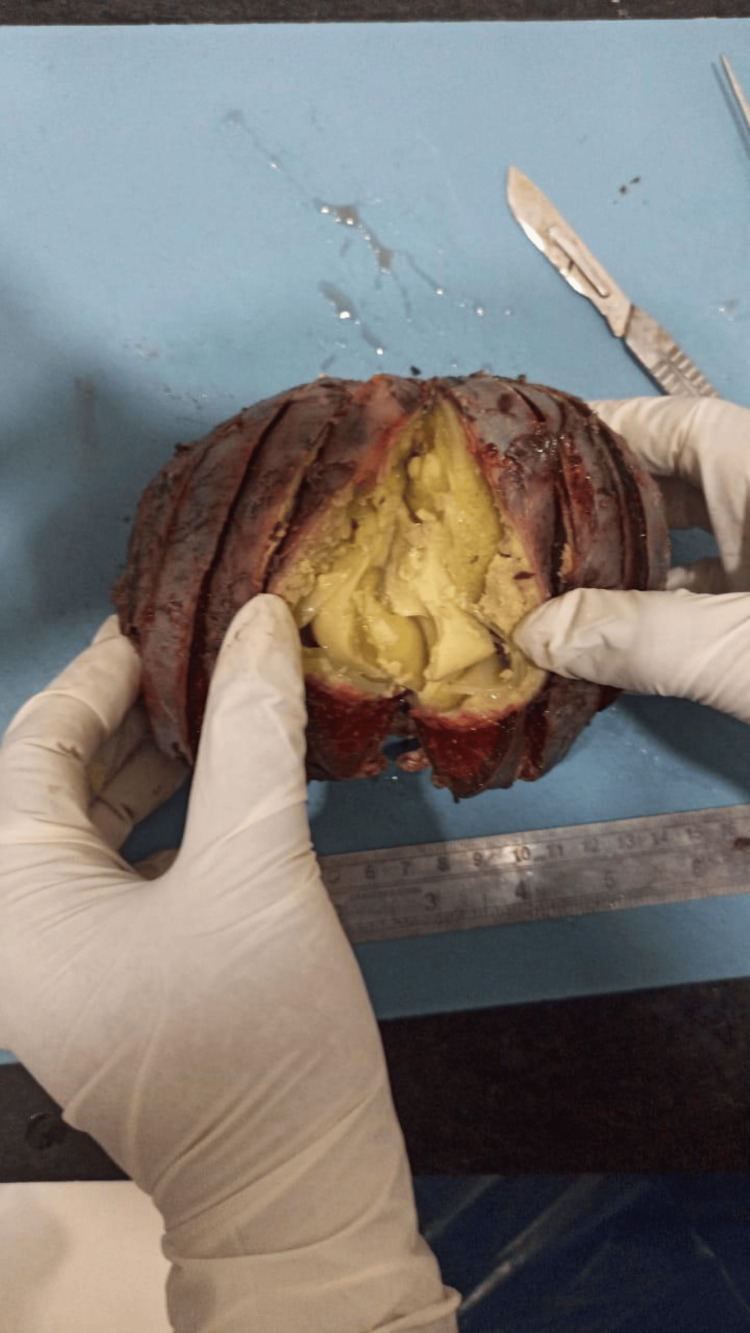
Entire splenic cavity covered by white cheesy material.

On the cut section, the entire splenic cavity was covered by white cheesy material (Figure 3). 

**Figure 4:**
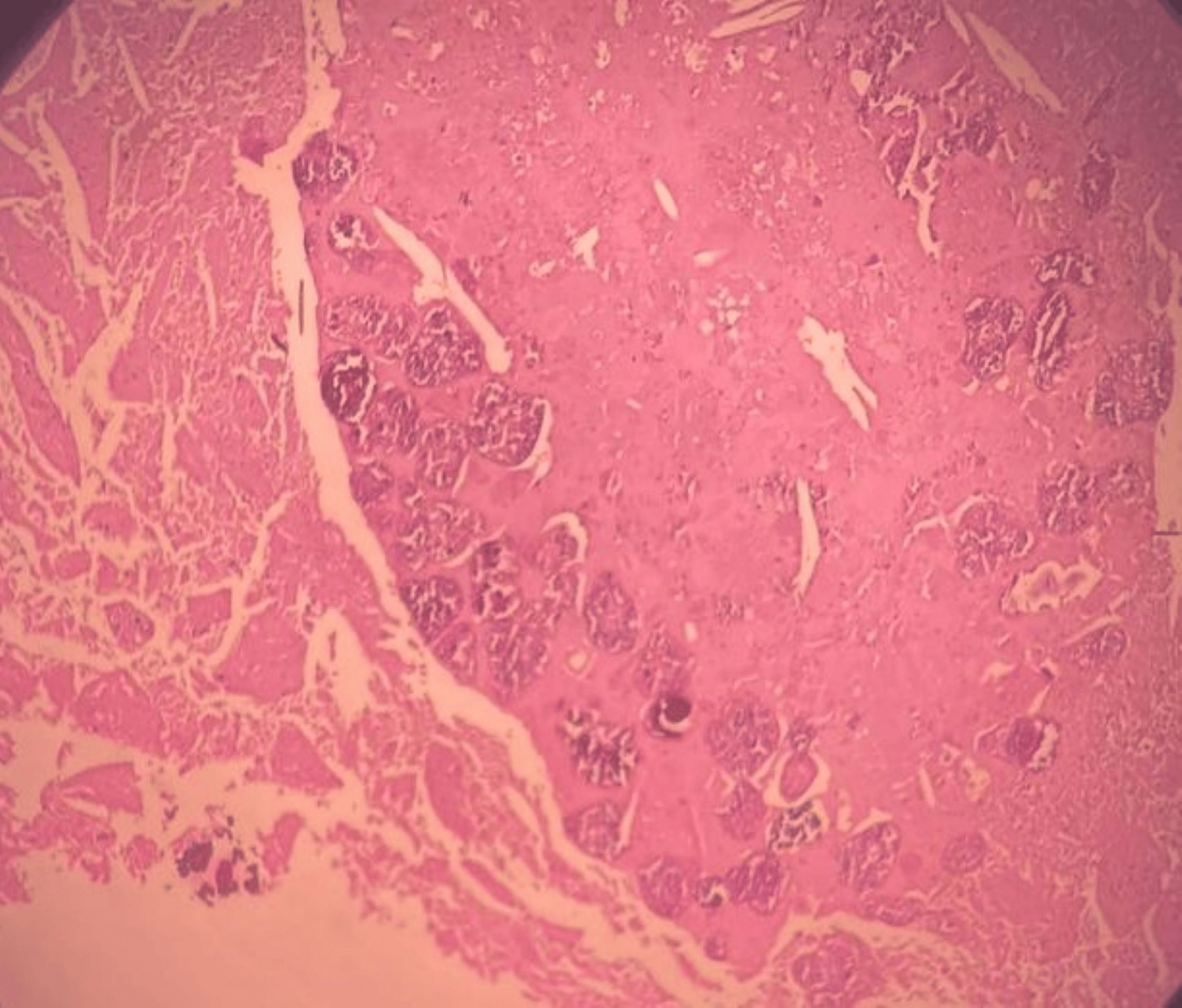
Section bits of laminated membrane, calcified foci and endocyst with scolices.

**Figure 5:**
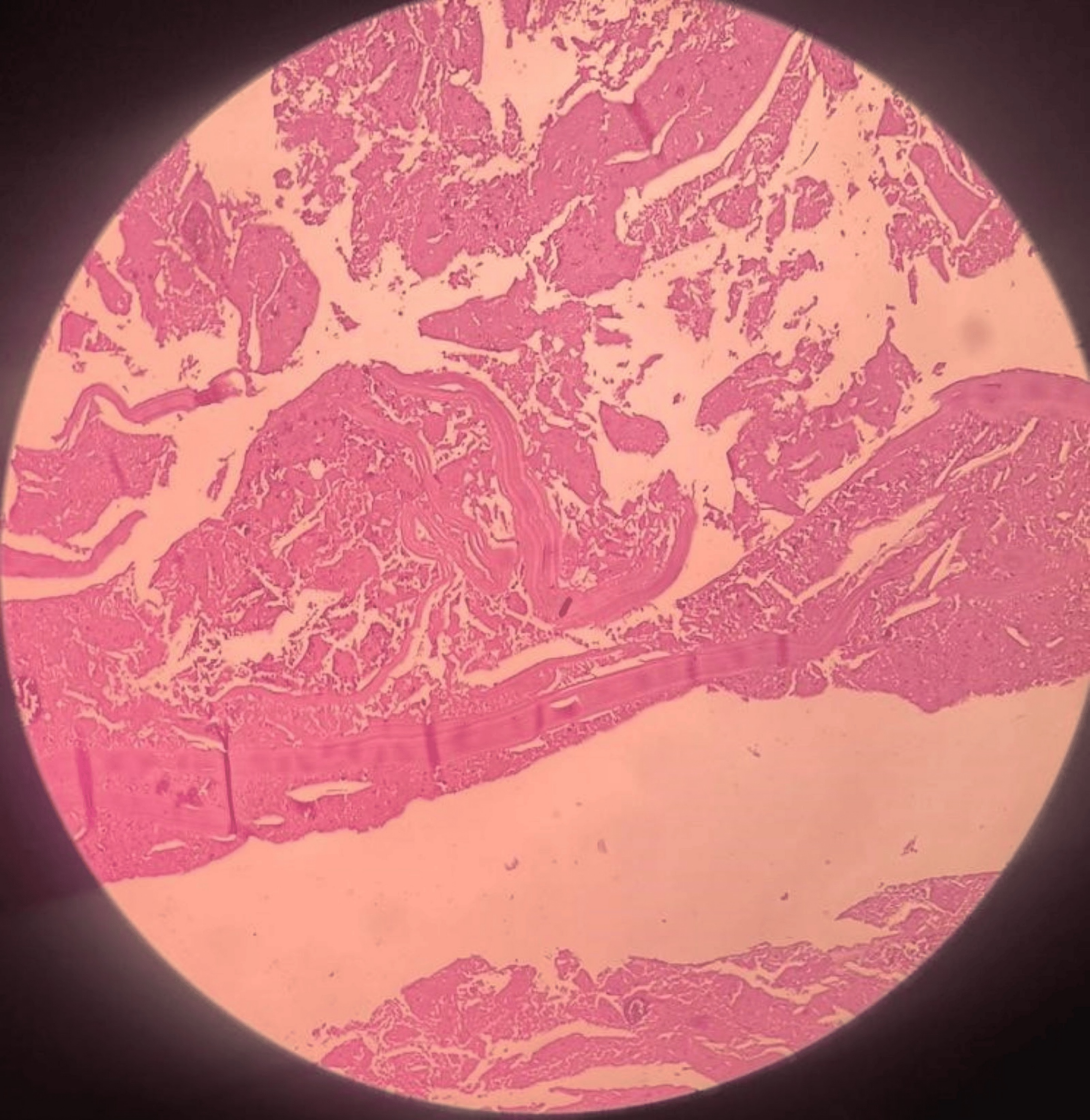
Sections showed a large area of spleen invaded by extensive fibrosis, surrounding bits of laminated membrane, calcified foci and chronic inflammatory infiltrate.

Along with the spleen, there were multiple irregular whitish tissue pieces measuring 1.2 x 01 x 0.7 cm that were labeled as laminated membranes known as the endocyst, which is the lining membrane of a hydatid cyst. On histopathology, sections showed a large area of spleen invaded by extensive fibrosis, surrounding bits of laminated membrane, calcified foci, and chronic inflammatory infiltrate (Figures 4, 5). The above-mentioned histopathological features were suggestive of the hydatid cyst of the spleen.

## Discussion

Hydatid disease, also known as echinococcosis or hydatidosis, is a form of parasitic infestation caused by a cestode, namely, *Echinococcus granulosus* (tapeworm). It is endemically seen in parts of the world that have agriculture or cattle breeding as a major occupation. The definitive host of the tapeworm in which the reproductive cycle takes place is the dog, with cattle or sheep being the intermediate hosts that are hosts to the non-reproductive forms or the immature forms of the tapeworm. Humans are incidental hosts that have no role to play in reproduction and are infested by either interaction with an already-infested dog or via contaminated food that has the eggs of the parasite [1]. Hydatid cysts are typically composed of three layers, which include pseudocyst (the outermost layer), ectocyst (middle layer of the laminated membrane), and endocyst (the innermost layer that secretes the hydatid fluid, leakage of which into the peritoneal cavity can lead to anaphylactic shock).

Usually, hydatid cysts do not show any major symptoms that would raise suspicion. The most commonly observed complaint of the patients is usually a dull aching pain that is not associated with any enhancing or alleviating factors. As the size of the cyst increases, the patient may develop pressure symptoms due to pressure exerted by the cyst on the left part of the diaphragm (causing breathlessness) or on the colon (causing changes in the pattern of bowel movements). Complications like anaphylactic shock can be seen in cases of rupture of the cyst, acute abdomen, splenic atrophy [3], and fatal massive hemoperitoneum [4].

On hematological investigations, there can be eosinophilia along with an increase in serum IgE levels. Radiological investigations such as ultrasonography, CT scan, and MRI are useful diagnostic modalities that may show the classic findings of a double-layered cyst wall along with daughter cysts [4]. The mainstay of treatment is surgery (splenectomy); however, vigilance while performing the surgery is of vital importance to avoid spillage of any contents of the cyst fluid to ensure there is no possibility of anaphylactic reactions. Splenectomy can be partial, subtotal, or total along with the puncture aspiration injection re-aspiration (PAIR) technique [5]. It is regarded as the first line of treatment for uncomplicated hydatid cysts [6]. For this technique, informed consent is obtained, followed by securing large intravenous access, readily available steroids, antihistaminics and adrenaline injections in case of the feared complication of anaphylactic shock in case of a mishap of cyst rupture [7]. 35% hypertonic saline is used as a scolicidal agent, and 95% absolute alcohol as the sclerosing agent [6]. Continuous monitoring with ultrasound is required while the procedure is being conducted. Shrinkage of the cyst or disappearance of the fluid is noted. Medical management includes the antihelminthic medication albendazole in the dosage of 400 mg twice a day. It is preferred to start treatment with albendazole at least four hours to one week prior to PAIR [8]. 

Complications of this technique include anaphylaxis, pneumothorax, and infection in the cystic cavity. Out of these, anaphylaxis is the most feared complication that may result in death due to vascular collapse and airway obstruction [9]. The risk for recurrence of hydatid cysts is low with PAIR, with lesser side effects and a low mortality rate [10].

Adlakha et al. [3] encountered a case of splenic hydatid cyst in a 46-year-old male, with primary complaints of only pain and a non-tender palpable mass in the left hypochondrium. CECT's abdomen showed a multiseptate cystic lesion suggestive of a splenic hydatid cyst. Post-surgical, the diagnosis was confirmed on histopathology.

Merad et al. [4] had a coincidental finding of a splenic hydatid cyst in a 28-year-old female. Biological confirmation of it was done by hemagglutination. Post-spleen-sparing surgery, the diagnosis was confirmed on histopathology. The patient was given albendazole and recovered adequately.

## Conclusions

Hydatid disease of the spleen is a zoonotic disease of rare occurrence. It is an accidental finding in most patients with no obvious symptoms other than dull dragging pain in the abdomen. It should be considered as a possible diagnosis in endemic areas that have agriculture or cattle breeding as a major occupation. Some patients may present with acute harrowing conditions like anaphylactic shock due to rupture of the cyst or massive hemoperitoneum. Diagnostic modalities are usually radiological investigations. Pre- and post-operative medical therapy includes albendazole and praziquantel. The mainstay of treatment is surgery.

To have encountered this case, with the patient having no presenting complaints except for pain in the abdomen, and finally concluding the diagnosis of hydatid cyst of the spleen, it is definitely a recherché experience to have been a part of the diagnostic team. Given the infrequent incidence of this, we hope to help fellow clinicians and pathologists in establishing a comparatively easier path for this diagnosis, given the lack of any classical symptoms other than a high index of suspicion.

## References

[1] Rasheed K, Zargar SA, Telwani AA (2013). Hydatid cyst of spleen: a diagnostic challenge. N Am J Med Sci.

[2] Meimarakis G, Grigolia G, Loehe F, Jauch KW, Schauer RJ (2009). Surgical management of splenic echinococcal disease. Eur J Med Res.

[3] Adlakha D, Chawla S, Utaal MS, Sharma S, Khurana A, Thakor M, Ramnani S (2024). Splenic hydatid cyst: a case report. Int Surg J.

[4] Merad Y, Derrar H, Zeggai A, Chadli M, Bemrah N, ElHabachi B (2021). Primary splenic hydatid cyst an unexpected diagnosis: case report. Ann Med Surg (Lond).

[5] Abdelmaksoud MM, Jamjoom A, Hafez MT (2020). Simultaneous huge splenic and mesenteric hydatid cyst. Case Rep Surg.

[6] Kahriman G, Ozcan N, Dogan S, Karaborklu O (2017). Percutaneous treatment of liver hydatid cysts in 190 patients: a retrospective study. Acta Radiol.

[7] Khuroo MS (2021). Percutaneous drainage in hepatic hydatidosis-the PAIR technique: concept, technique, and results. J Clin Exp Hepatol.

[8] Bhalla VP, Paul S, Klar E (2023). Hydatid disease of the liver. Visceral Med.

[9] Atay G, Erdogan S, Tugrul HC, Su Dur SM (2023). Anaphylaxis during puncture of a hepatic hydatid cyst. North Clin Istanbul.

[10] Fayyaz A, Ghani UF (2013). Successful treatment of hydatid cyst of lesser sac with PAIR therapy. J Coll Physicians Surg Pak.

